# Blood Pressure and Risk of Subarachnoid Hemorrhage in China

**DOI:** 10.1161/STROKEAHA.118.022239

**Published:** 2018-12-07

**Authors:** Iain John McGurgan, Robert Clarke, Ben Lacey, Xiang Ling Kong, Zhengming Chen, Yiping Chen, Yu Guo, Zheng Bian, Liming Li, Sarah Lewington

**Affiliations:** 1 From the Nuffield Department of Population Health (I.J.M., R.C., B.L., X.L.K., Z.C., Y.C., S.L.), University of Oxford, United Kingdom; 2 Medical Research Council Population Health Research Unit, Nuffield Department of Population Health (B.L., X.L.K., S.L.), University of Oxford, United Kingdom; 3 Chinese Academy of Medical Sciences, Beijing, China (Y.G., Z.B., L.L.); 4 Department of Epidemiology, School of Public Health, Peking University Health Science Center, Beijing, China (L.L.).

**Keywords:** blood pressure, China, humans, risk factors, subarachnoid hemorrhage

## Abstract

**Background and Purpose—:**

Subarachnoid hemorrhage (SAH) has a high case fatality rate and young mean age at onset compared with other types of stroke, but the pathogenesis of SAH is not fully understood. We examined associations of systolic and diastolic blood pressure with incident nontraumatic SAH in a large prospective study in China.

**Methods—:**

In 2004 to 2008, 512 891 adults (59% women) from the general population were recruited into the CKB study (China Kadoorie Biobank). Participants were interviewed, measured, and followed up for fatal and nonfatal events. After excluding those with prior vascular disease, Cox regression analysis was used to relate blood pressure to incident SAH events. Analyses were adjusted for major confounders and corrected for regression dilution to give associations with long-term average blood pressure.

**Results—:**

At baseline, mean age was 51 (SD, 11) years, and mean systolic blood pressure/diastolic blood pressure was 130.6/77.6 (SD, 21.0/11.1) mm Hg. During 3.5 million person-years of follow-up, there were 553 incident SAH cases (mean age at event, 61 [SD, 11] years), yielding an overall annual incidence rate of 12.9 per 100 000. Higher average levels of blood pressure were linearly and positively associated with higher risks of incident SAH: a 10 mm Hg higher systolic blood pressure and a 5 mm Hg higher diastolic blood pressure were associated with hazard ratios for SAH of 1.21 (95% CI, 1.13–1.29) and 1.20 (95% CI, 1.12–1.28), respectively. There was no evidence that the hazard ratios varied by age or sex or by levels of other vascular risk factors. Elevated blood pressure (systolic blood pressure, >120 mm Hg) accounted for 23% of all SAH cases.

**Conclusions—:**

The incidence of SAH in China was comparable with estimates from Western populations. Higher levels of blood pressure were positively associated with higher risks of SAH, and elevated blood pressure accounted for about a quarter of all SAH cases.

Subarachnoid hemorrhage (SAH) is less common than ischemic stroke and intracerebral hemorrhage, but it has a substantial impact on population health because of its high case fatality rate and young mean age at onset.^1^ The incidence of SAH has been found to vary substantially worldwide, with particularly high rates in Finland and Japan.^2^ Previous studies have suggested lower incidence rates of SAH in China compared with Western populations or with other Asian populations, but the precise reasons for such differences are not fully understood.^3–6^

Established risk factors for spontaneous (nontraumatic) SAH include elevated blood pressure, smoking, and high alcohol intake.^7,8^ The relatively low incidence of SAH in China is surprising given the high prevalence of such risk factors in Chinese adults, particularly in men. Overall, about one-third of Chinese adults have hypertension, but only one-third of those are diagnosed, and of those, only half are treated.^9^ Meta-analyses of prospective studies reported that hypertension is associated with about a 2-fold higher incidence of SAH.^10,11^ However, the effects of blood pressure on SAH risk in China have not been well established and may differ from those in other parts of the world.

Reliable assessment of the incidence of SAH and its association with blood pressure requires large prospective studies with ascertainment of both nonfatal and fatal events, avoidance of misclassification between stroke types (requiring high rates of brain imaging), and repeat measures of blood pressure (to allow correction for within-person variability, which results in regression dilution bias). The aims of the present report were to use the CKB study (China Kadoorie Biobank) of 0.5 million adults to (1) estimate the age- and sex-standardized incidence of SAH in the study population; (2) assess the shape and strength of the associations of blood pressure with risk of SAH after adjustment for major confounders and correction for regression dilution; and (3) determine the proportion of all SAH cases because of elevated blood pressure.

## Methods

### Baseline Survey

The CKB study is a prospective study of adults aged 30 to 79 years who were recruited from 10 diverse areas (5 urban and 5 rural) of China in 2004 to 2008.^12^ Participants were identified through public registry records and invited to attend a local assessment center. Information on prior medical history, lifestyle factors, and use of medication were collected using interviewer-administered electronic questionnaires. Measurements of blood pressure, weight, and height were also recorded on all participants at baseline. Ethics approval was obtained from the relevant local, national, and international ethics committees, and all participants provided written informed consent. Data from the baseline survey, first resurvey, and cause-specific mortality are available to all bona fide researchers (www.ckbiobank.org). Additional data may be made available on a collaborative basis by contacting the study investigators.

### Measurement of Blood Pressure

Blood pressure was measured by trained staff with a UA-779 digital sphygmomanometer (A&D Instruments) using standardized procedures at all study sites. Measurements were recorded twice, after at least 5 minutes in a seated position, and the mean of both the measurements of systolic blood pressure (SBP) and diastolic blood pressure (DBP) was recorded. If the values of SBP differed by >10 mm Hg, a third measurement was recorded, and the mean of the last 2 measurements was used.^12^

### Resurvey and Follow-Up

In 2008, a resurvey was performed of ≈5% of participants selected using cluster random sampling, with administrative units (rural villages or urban residential committees) as the sampling units, using the same procedures as those for the baseline survey. Repeat blood pressure measures were used to correct the prospective analyses for regression dilution to give associations with long-term average (usual) blood pressure. Deaths were identified by electronic linkage to local records of the Disease Surveillance Points system of the Chinese Center for Disease Control and Prevention. Nonfatal events were collected using linkage to stroke registries, mortality registries, and health insurance records. All fatal and nonfatal events were coded using the *International* *Statistical Classification of Diseases and Related Health Problems, Tenth Revision* (SAH [I60]); >90% of incident stroke cases had confirmation of diagnosis by computed tomography/magnetic resonance brain imaging. Residential records were used to identify participants who moved out of the study regions and, by so doing, were lost to follow-up. By January 1, 2016, 37 289 (7.3%) participants had died and only 4875 (1%) participants were lost to follow-up.

### Statistical Analysis

Analyses excluded participants with missing information for blood pressure or other key variables (n=2), individuals with SBP <80 or ≥250 mm Hg (n=116) or with DBP <40 or ≥150 mm Hg (n=62), and individuals with a prior history of vascular disease (stroke, transient ischemic attack, or coronary heart disease: n=23 129), leaving 489 613 participants for the present analyses. After these exclusions, the incidence rates of SAH were calculated and standardized to the age and sex distribution of the study population. Cox regression analysis^13^ was used to estimate hazard ratios (HRs) for the associations of blood pressure with SAH incidence, with adjustment for age at risk (5-year age groups), sex, area, education, smoking, alcohol intake, and body mass index.

The HRs per 10 mm Hg higher SBP were obtained by fitting SBP as a continuous variable in men and women separately for each decade of age at risk (<50, 50–59, 60–69, and 70+ years).

The combined effects of random measurement errors and within-person fluctuations of blood pressure over time will underestimate the associations of blood pressure recorded at baseline with incidence of SAH.^11^ Repeat measurements of blood pressure in random samples of participants were used to correct for this regression dilution bias.^14^ The amount by which the true association was underestimated by using baseline instead of long-term average (usual) values of the risk factor of interest was expressed as the regression dilution ratio. Analyses of the associations of SAH with blood pressure were corrected for regression dilution by using the appropriate regression dilution ratios for each age- and sex-specific group. Hence, age- and sex-specific HRs were then corrected for age- and sex-specific regression dilution ratios (using estimates from the slope of the linear regression between baseline and resurvey blood pressure values^14^) to give HRs for 10 mm Hg higher usual SBP.

In categorical analyses, baseline blood pressure was divided into the following groups: 80 to <125, 125 to <145, 145 to <165, 165 to <185, and 185 to <250 mm Hg for SBP and 40 to <75, 75 to <85, 85 to <95, 95 to <105, and 105 to ≥150 mm Hg for DBP. HRs were estimated relative to the lowest blood pressure groups, and the 95% CIs were calculated using the variance of the log risk. HRs were plotted against the mean SBP or DBP in each group, with the effect of increasing the slope of association by a factor equal to the inverse of the regression dilution ratio.

Assuming causality, the fraction of SAH cases attributable to elevated blood pressure in each blood pressure group was estimated by P_d_×(relative risk−1)/relative risk, where P_d_ represents the proportion of all SAH cases in a given blood pressure group (using the same blood pressure groups as described above), and relative risk is approximated by the group-specific HRs (this approximation is valid early in follow-up, when the event rate is low) relative to the lowest blood pressure group.^15^ The overall fraction of SAH cases attributable to blood pressure was calculated as the sum of the fractions in each of the blood pressure groups. All analyses used SAS software (version 9.3), and Figures were plotted using R software (version 3.3).

## Results

Between June 2004 and July 2008, 512 891 adults were recruited, yielding an overall response rate of ≈30% of the resident population in each of the areas studied. After exclusions, 489 613 participants remained. Mean age at baseline survey was 51 (SD, 11) years, and 59% of participants were women (Table 1). Overall, 49% had been educated to middle school or higher, and 43% were from urban areas. Among men, 62% were current smokers and 34% consumed alcohol at least weekly, but only 2% of women were smokers and 2% consumed alcohol at least weekly. Mean SBP was 130.6 (SD, 21.0) mm Hg, and mean DBP was 77.6 (SD, 11.1) mm Hg, and 4% reported taking blood pressure-lowering medication. At baseline, SBP increased linearly with age in both men and women, but DBP increased with age up until 50 years of age, after which it declined. Mean levels of SBP were inversely associated with levels of education and living in an urban area and positively associated with body mass index, higher alcohol intake, and diabetes mellitus.

**Table 1.**
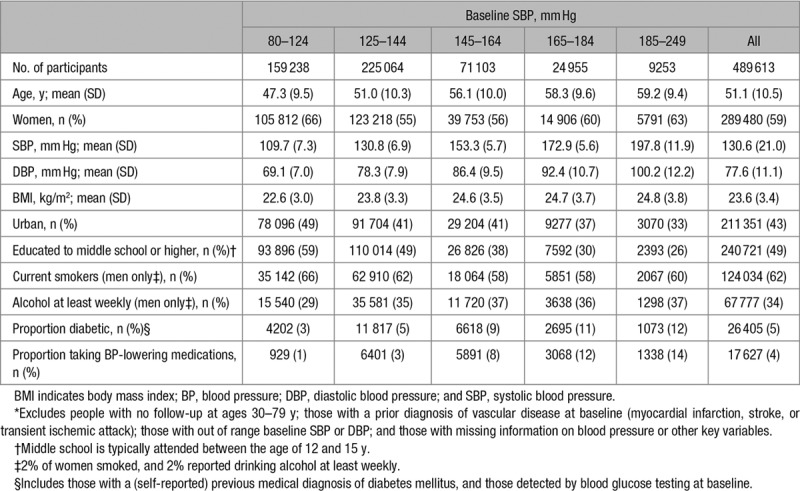
Baseline Characteristics of the 489 613 Participants in the Main Analyses,* by SBP

Among the 18 836 participants with baseline and resurvey data, the demographic characteristics were comparable with those in the overall population. The mean time from baseline to the first resurvey was 2.6 (SD, 0.7) years. Overall, the weighted mean regression dilution ratios for SBP and DBP were 0.58 and 0.58, respectively (albeit these were slightly more extreme in older versus younger age groups and in men compared with women; Table 2).

**Table 2.**
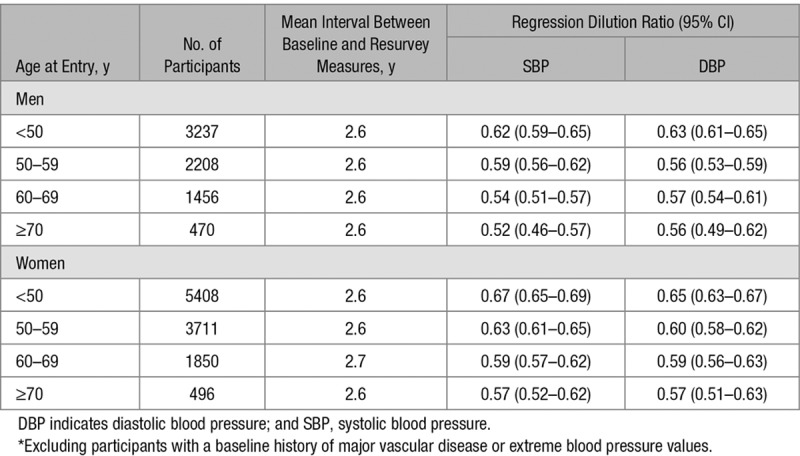
Age- and Sex-Specific Regression Dilution Ratios for SBP and DBP in 18 836 Participants With Baseline and Resurvey Data*

During 3.5 million person-years of follow-up, there were 553 incident cases of SAH, yielding an overall incidence rate of 12.9 per 100 000. The incidence rates increased substantially with age in both sexes, and the age-standardized rates were slightly higher in women than in men (13.5 versus 11.9 per 100 000). In both men and women, the incidence rates were lower in urban than in rural areas (9.9 versus 14.5 per 100 000), although these differences were more marked in men. The mean age at incident SAH event was 61 (SD, 11) years. Separate incidence rates are provided for individuals classified by smoking, drinking, overweight, and use of antihypertensive medication at baseline.

**Figure 1.**
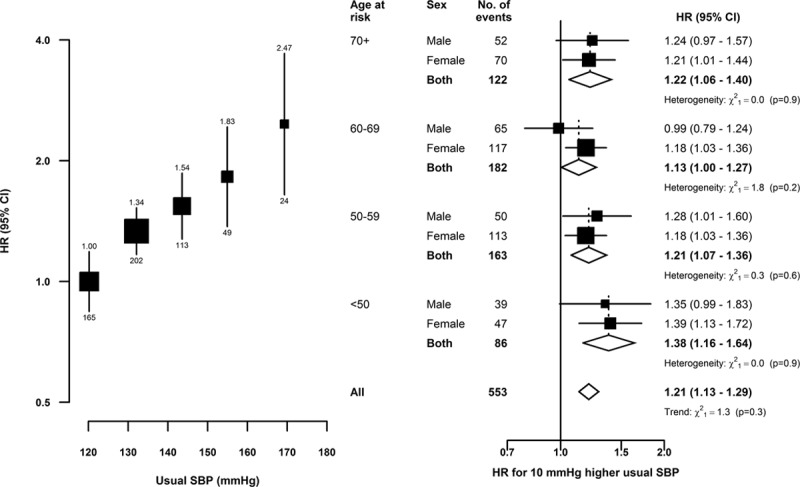
Subarachnoid hemorrhage vs usual systolic blood pressure (SBP), by age and sex. Hazard ratios (HR) adjusted for age at risk, sex, area, education, smoking, alcohol intake, and body mass index. **Left**, the area of each square is inversely proportional to the variance of the category-specific log risk; (**right**) it is inversely proportional to the variance of the log(HR). Corresponding 95% CIs are plotted as lines. Each diamond is the inverse-variance weighted average of the 2 estimates for men and women. Regression dilution ratios: (**left**) 0.58; (**right**) by sex and age, for men: 70+ (0.52), 60−69 (0.54), 50−59 (0.59), and <50 (0.62) y, and for women: 70+ (0.57), 60−69 (0.59), 50−59 (0.63), and <50 (0.67) y.

**Figure 2.**
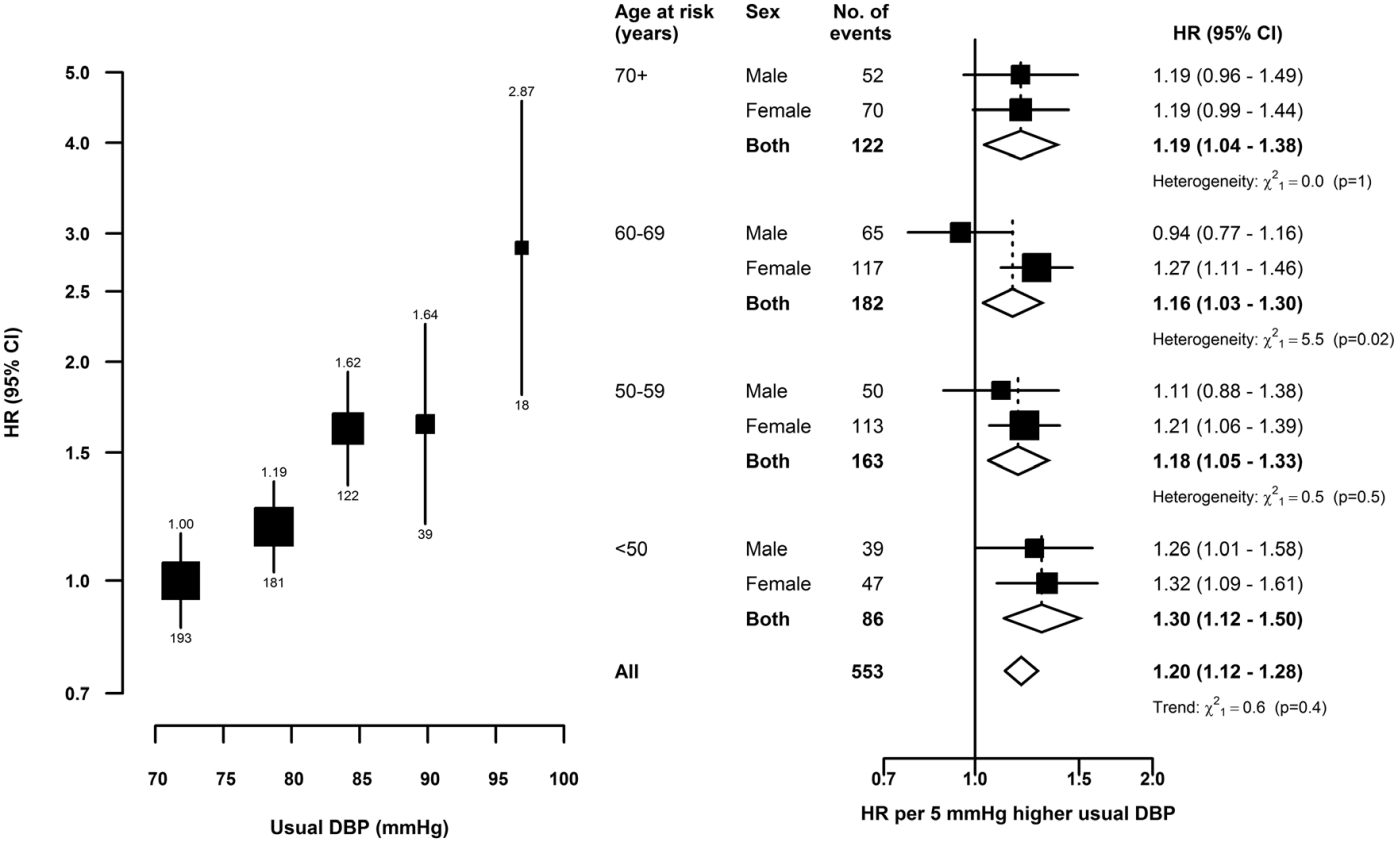
Subarachnoid hemorrhage vs usual diastolic blood pressure (DBP), by age and sex. Symbols and conventions as in Figure 1. Regression dilution ratios: (**left**) 0.58; (**right**) by sex and age, for men: 70+ (0.56), 60−69 (0.57), 50−59 (0.56), and <50 (0.63) y, and for women: 70+ (0.57), 60−69 (0.59), 50−59 (0.60), and <50 (0.65) y.

**Figure 3.**
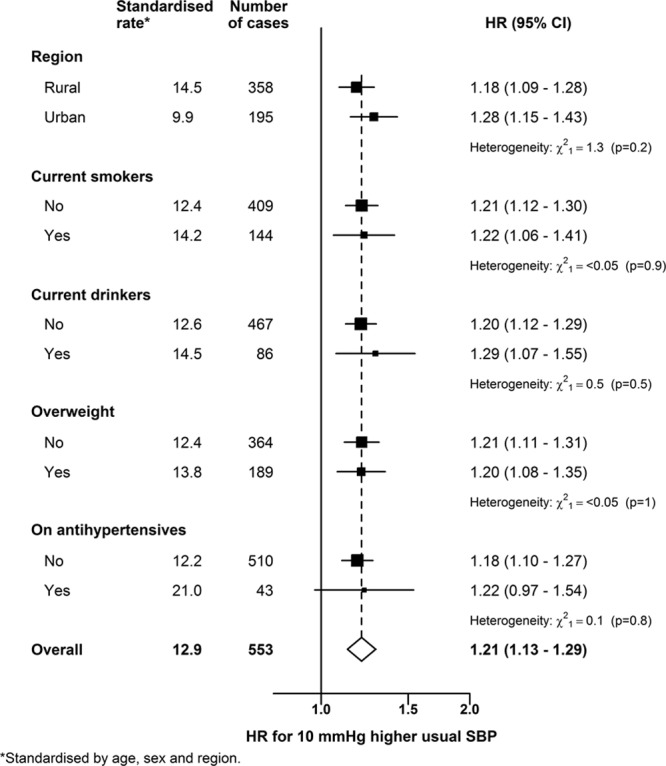
Hazard ratios (HR) for subarachnoid hemorrhage associated with a 10 mm Hg higher usual systolic blood pressure (SBP) in relevant subgroups HR are shown for region (rural/urban), current smokers (no/yes), current drinkers (no/yes), overweight (no/yes), and use of antihypertensive medication (no/yes). Symbols and conventions as in Figure 1.

Higher usual levels of SBP were linearly and positively associated with risk of SAH throughout the range of SBP values studied (Figure 1). After correction for regression dilution, a 10 mm Hg higher usual level of SBP was associated with 21% (95% CI, 13–29) higher risk of SAH. Likewise, DBP was also linearly and positively associated with SAH, and the strength of the association for a 5 mm Hg higher usual level of DBP was similar to that for a 10 mm Hg higher usual level of SBP (Figure 2). There was no evidence of effect modification of these associations by age nor of the age-specific associations by sex. Furthermore, there was no evidence of effect modification of the associations of SAH with SBP by area (rural/urban), smoking, drinking, overweight (body mass index, ≥25 kg/m^2^), or use of antihypertensive medication at baseline (Figure 3). Assuming causality, the excess SAH incidence associated with elevated blood pressure (ie, SBP >120 mm Hg) accounted for 23% (95% CI, 14–30) of all SAH events.

## Discussion

In this Chinese population, with a mean age at baseline of 51 years, the overall incidence of SAH was 12.9 per 100 000. Higher levels of blood pressure were linearly and positively associated with higher risks of SAH incidence throughout the range of blood pressures examined. Overall, a 10 mm Hg higher usual level of SBP, or a 5 mm Hg higher level of DBP, was associated with about 20% higher risk of SAH. There was no evidence that the strength of these associations varied by age or sex or by level of other established vascular risk factors. It was estimated that 23% of all SAH cases were attributable to elevated blood pressure.

The incidence rates of SAH observed in the present study were higher than those reported by previous studies conducted in China^3,16,17^ but similar to those in Western populations.^2^ The present study used several complementary methods to ascertain cases, including linkage to stroke and mortality registries, together with linkage to health insurance records for all hospitalizations, which should have identified almost all the SAH cases in the study population.

In general, the factors contributing to higher prevalence of SAH include a predisposition to intracranial aneurysm rupture in certain populations^18^ and a high prevalence of hypertension, smoking, and excessive alcohol consumption.^8^ Mean levels of blood pressure vary widely by geographic region in China,^19^ and the design of the CKB study could account for slightly higher incidence rates compared with previous studies conducted in more limited geographic distributions of China. However, it is also possible that some of the higher incidence of SAH compared with previous studies in China could reflect changes in risk factor profiles, including a higher prevalence of hypertension, smoking, and physical inactivity.^20–23^

The shape and strength of the associations of levels of blood pressure with the risk of SAH demonstrated in the present study were consistent with the results of previous studies conducted in Western populations.^10,11,24–26^ These findings reinforce the importance of public health strategies to reduce elevated blood pressure in China, including the increased use of blood pressure-lowering medication in clinical practice, and public health initiatives to reduce salt intake, high alcohol intake, excess adiposity, and poor home heating.^19,27,28^

The strengths of this study include its large sample size, the standardized procedures for assessment of blood pressure, adjustment for confounding, and minimization of bias. Selection bias was limited by the broad inclusion criteria for participation and by low (<1%) rates of attrition.^29^ Reverse causality bias was minimized by the exclusion of participants with a prior history of vascular disease. Regression dilution ratios derived in this study were comparable with estimates obtained in previous studies.^10,11^ Potential limitations of the present study include the relatively small number of SAH cases in some subgroups and the possibility of residual confounding by unmeasured participant characteristics, such as plasma lipid concentrations, or family history of vascular disease.

## Conclusions

The incidence rates of SAH in China were higher than those reported in previous studies in Chinese populations and are consistent with worldwide estimates. The present study demonstrated strong, positive linear associations of levels of blood pressure with risk of SAH among Chinese adults. The shape and strength of these associations were comparable with those reported in previous prospective studies in Western populations. These findings have important implications for the prevention of SAH in China. Interventions producing moderate population-wide reductions in blood pressure should result in substantial reductions in the burden of SAH. Based on both the high loss of productive life years by SAH and the increasing prevalence of hypertension in China, the importance of such interventions cannot be understated.

## Acknowledgments

The chief acknowledgment is to the participants, the project staff, and the China National Center for Disease Control and Prevention and its regional offices for access to death and disease registries. The Chinese National Health Insurance scheme provides electronic linkage to all hospital admission data.

## Sources of Funding

The CKB study (China Kadoorie Biobank) is jointly coordinated by the University of Oxford and the Chinese Academy of Medical Sciences. The funding body for the baseline survey was the Kadoorie Charitable Foundation, Hong Kong, China, and the funding resources for the long-term continuation of the study include UK Wellcome Trust (202922/Z/16/Z, 104085/Z/14/Z, and 088158/Z/09/Z), Chinese National Natural Science Foundation (81390540, 81390541, and 81390544), and the National Key Research and Development Program of China (2016YFC0900500, 2016YFC0900501, 2016YFC0900504, and 2016YFC1303904). Core funding was provided to the Clinical Trial Service Unit, University of Oxford, by the British Heart Foundation, UK Medical Research Council, and Cancer Research UK. B. Lacey acknowledges support from the National Institute for Health Research Oxford Biomedical Research Centre. The funders had no role in study design, data collection and analysis, decision to publish, or preparation of the manuscript.

## Disclosures

None.
